# Impact of Pegylated Interferon-alfa-2a on Perforin Level in Patients With Chronic Hepatitis B; Preliminary Study

**DOI:** 10.5812/hepatmon.11903

**Published:** 2013-11-23

**Authors:** Meisam Mahdavi, Houshang Amirrasouli, Seyed Moayed Alavian, Bita Behnava, Faranak Kazerouni, Maryam Keshvari, Saeed Namaki, Mohammad Gholami Fesharaki, Hooman Rahimipour, Jahangir Mohammadzade, Farahnaz Zohrehbandian, Fazel Mahdavipour

**Affiliations:** 1 Department of Laboratory Sciences, Faculty of Paramedical Sciences, Shahid Beheshti University of Medical Sciences, Tehran, IR Iran; 2 Baqiyatallah Research Center for Gastroenterology and Liver Diseases, Baqiyatallah University of Medical Sciences, Tehran, IR Iran; 3 Middle East Liver Diseases Center (MELD Center), Tehran, IR Iran; 4 Blood Transfusion Research Center, High Institute for Research and Education in Transfusion Medicine, Tehran, IR Iran; 5 Biostatistic Department, Faculty of Medicine, Tarbiat Modares University, Tehran, IR Iran; 6 Faculty of Medicine, Shahid Beheshti University of Medical Sciences, Tehran, IR Iran; 7 Department of Microbiology, Faculty of Basic Sciences, Islamic Azad University, North Tehran Branch, Tehran, IR Iran; 8 Faculty of Medicine, Ilam University of Medical Sciences, Ilam, IR Iran

**Keywords:** Hepatitis B, Perforin, PEG-IFN-Alfa-2a

## Abstract

**Background:**

Chronic hepatitis B is one of the most common causes of cirrhosis and hepatocellular toxicity in many countries, including Iran. Cytotoxic T lymphocyte (CTL) and Natural killer (NK) cells are the two of main cell populations considered as cytotoxic cells. One of the distinct pathways CTL and NK cells exert cytotoxicity is perforin/granzyme. After the cytotoxic cell/target cell junction, perforin is released from granules by exocytosis. Once it is anchored, perforin forms cylindrical pores through which granzymes and granulysin enter and induce apoptosis.

**Objectives:**

Large controlled trials have demonstrated the efficacy of PEG-IFN-α-2a in treatment of chronic hepatitis B. This study was aimed to examine whether the enhancement of cytotoxicity by PEG-IFN-α-2a is mainly due to the perforin pathway.

**Patients and Methods:**

This research work was performed on 50 patients and five healthy people. Patients with chronic hepatitis B were further subdivided into two groups: patients with inactive chronic hepatitis B (carriers, n = 30), and those with active chronic hepatitis B who were under treatment with PEG-IFN-alfa-2a (n = 20) for minimum six and maximum 12 months. Serum perforin level was measured using ELISA method (CUSABIO Company), HBV viral load was assessed using COBAS Taq-man, and we used Elecsys hepatitis B surface antigen (HBs Ag) II quantitative assay method for HBs Ag determination. HBeAg was evaluated by ELISA method, and AST and ALT were measured by routine laboratorymethods.

**Results:**

Based on the results obtained serum perforin level in healthy group was 0.64 ng/mL, the mean of serum perforin level in inactive HBs Ag carriers was 2.63ng/mL, and 4.63 ng/mL in patients with active chronic hepatitis B under treatment with PEG-IFN-α-2a. The mean of serum perforin level in patients with and without virologic response to treatment were 5.45 ng/mL,and 3.4 ng/mL respectively. Finally in patients with virologic response and seroconverted serum perforin level was 7.23 ng/mL.

**Conclusions:**

Based on our results higher perforin level in patients under treatment with PEG-IFN-α-2a, could be an indication of elevated cytotoxicity via perforin/granzyme pathway.

## 1. Background

Cytotoxic T cell (CTL) and natural killer cell (NK cell) are indispensable factors in the body ongoing defense against viral infection (1). CTL and NK cell recognize and kill infected or aberrant target cells, the predominant pathway for CTL and NK cell induced cell death is often the granule mediated pathway (2). Granule–dependent exocytosis pathway is performed by intracellular signaling after recognition by cytotoxic lymphocyte (NK cell or cytotoxic Tcell) (3). The granules that induce apoptosis contain lytic molecules such as perforin, granzymes (Grzs), and granulysin (4). In this pathway, cytoplasmic granule toxins, predominantly perforin, and a family of structurally played serine proteases (granzymes) are secreted by exocytosis (3, 5). Perforin is found in a soluble monomer shape within granules offer the cytotoxic cell/target cell junction. Once it is anchored, perforin begins polymerization in the presence of Ca^2+^, forming cylindrical pores through which granzymes and granulysin enter and induce apoptosis within virus infected cells, and thus destroy them (5, 6). Several agents currently are approved for the treatment of chronic hepatitis B: interferon (IFN) alfa-2b, pegylated interferon (PEG IFN) alfa-2a, and antiviral agent such as lamivudine, adefovir and etc. IFNs exert an antiviral effect on HBV infection through two mechanisms: first a direct antiviral effect inhibiting the synthesis of viral DNA and by activating antiviral enzymes, and a second mechanism which increases the cellular immune response against infected hepatocytes with HBV (7, 8). Recently the efficacy of IFN has been improved by attaching a large branched 40 KD polyethylene glycol molecule to interferon alfa and made PEG-IFN-alfa-2a (7).

## 2. Objectives

This study aimed to examine whether serum perforin level is affected by treatment with PEG-IFN-alfa-2a.

## 3. Patients and Methods

### 3.1. Patients

This research work was performed on 50 patients and 5 healthy volunteers. Patients with chronic hepatitis B were further subdivided into two groups: patients with inactive chronic hepatitis B (carriers, n = 30), and those with active chronic hepatitis B who were under treatment with PEG-IFN-alfa-2a (n = 20) for minimum six and maximum 12 months. PEG-IFN-alfa-2a was administrated 180 µg weekly by subcutaneous route in patients with active chronic hepatitis B.

The inactive HBs Ag carrier state is diagnosed by absence of HBeAg and presence of anti-HBe, undetectable or low levels of HBV DNA in PCR-based assays, repeatedly normal ALT levels, and minimal or no necroinflammation, slight fibrosis, or even normal histology on biopsy, and is considered to be associated with a favorable prognosis (9, 10). Chronic active patient with Hepatitis B infection is defined aspositive results for HBs Ag longer than 6 months, HBeAg positive findings, anti-HBe negative results, and serum HBV DNA greater than 105 copies/mL, and elevated or normal hepatic aminotransferase levels (11). However, absence or presence of HBeAg is not an absolute criterionfor reflection of activity or inactivity in HBV infection (10, 11).

Treatment response is defined as the loss of HBeAg with a serum HBV level below 2000 Copies/mL (400 IU/mL), and normal ALT levels (14). And in patients with negative results for HBeAg, virologic response has been defined as HBV DNA less than 2000 IU/mL six months after beginning the treatment (12).

In this study perforin and 6 parameters were assayed, methods of measurement are explained as below:

### 3.2. Perforin

In this study serum perforin level was measured in all participants; in patients with active chronic hepatitis B who were under treatment with PEG-IFN-alfa-2a for minimum 6 and maximum 12 months it was measured once during the treatment. Perforin was measured using ELISA method (CUSABIO Company). Perforin level is expressed in ng/mL, and the detection range is 0.312 ng/mL – 20 ng/mL.

### 3.3. HBV Viral Load

This parameter was assessed using COBAS Taq-man PCR (Fully automated hepatitis B virus (HBV) viral load quantitative for improved testing in serum and plasma) which is one of the best methods for assaying this parameter. Lower limit of detection for COBAS Taq-man PCR is 6 IU/mL equivalent to 35 Copies/ mL, COBAS Taq-man PCR method is the only FDA approved quantitative method for existing Hepatitis B in Iran. Measurement of viral load in this research work has been performed in all patients with chronic hepatitis B, and pre and post treatment in patients with active hepatitis B. In this study the scale of HBV viral load was IU/mL.

### 3.4. HBs Ag Quantitative

HBs Ag was determined in all patients using Elecsys hepatitis B surface antigen (HBsAg) II quantitative assay, a new quantitative electro chemiluminescence immunoassay which uses onboard dilution and a simple algorithm to determine HBsAg levels expressed in international units (IU)/mL (standardized against the World Health Organization [WHO] Second International Standard). Detection limit for this test is 5 IU/mL.

### 3.5. HBeAg

This parameter was evaluated by ELISA method (DIAPRO Company). HBeAg ELISA adopts the "sandwich principle" as the basis of evaluation. This parameter has been determined in all patients with chronic Hepatitis B; in patients with active chronic hepatitis B under treatment with PEG-IFN-alfa-2a it was determined pre and post treatment. If the HBeAg is cleared and anti-HBe appears, it is an indication of significant decline in virus replication (13). This parameter is one of the main standards for response to the PEG-IFN-α-2a treatment (8).

### 3.6. AST and ALT Serum Levels

The serum ALT and AST levels rise in patients with chronic hepatitis B which is observed in patients with active chronic hepatitis B especially (14). AST and ALT were measured by Hitachi 912 auto analyzer using diagnostic kit manufactured by Bionik Company. The level of these parameters has been expressed in international units (IU)/mL.

### 3.7. Stage and Grade of Disease

The Hepatitis B infection based on development of disease and rate of damage in liver is ranked in six stages and eighteen grades. Stage and grade in patients with active chronic Hepatitis B have been set by liver biopsy and pathological analyzing. The method for describing this parameter is modified by the Knodell’s scoring system.

### 3.8. Criteria for Response to Treatment

Aspects of response to treatment in this study were virologic response and seroconversion after 6 to 12 months in patients under treatment. The criteria for virologic response is viral load less than 2000 IU/mL, and for Seroconversion response is defined as disappearance of HBeAg, and appearance of anti-HBe.

### 3.9. Data Analysis

The descriptive statistics were provided with mean ± Standard Deviation (SD) or No. (%) as appropriate. χ2 and Fisher’s exact tests were used for comparing categorical data, and t-student test and Mann-Whitney U-test were used for analyzing continues variables. Spearman correlation was performedto correlate continuous variables. P values less than 0.05 were considered as statistically significant. Data was analyzed with SPSS version 18.

## 4. Results

50 patients with chronic hepatitis B and 5 healthy volunteers were included in this study. Patients with chronic hepatitis B were stratified into two groups: patients with inactive chronic hepatitis B (carriers, n = 30), and those with active chronic hepatitis B who were under treatment with PEG-IFN-α-2a (n = 20) for minimum six and maximum 12 months. It was shown that after treatment of 20 patients with active chronic hepatitis B, 14 had negative results for HBeAg pre and posts treatment, four patients were HBeAg positive pre and post treatment were as HBeAg seroconversion occurred in 2 patients receiving PEG-IFN-α-2a. In our study serum perforin level was significantly higher in patients than healthy subjects (Table 1). Based on our results perforin level was lower in the patients with inactive chronic hepatitis B compared to those with active chronic hepatitis B under treatment with PEG-IFN-α-2a (Table 1). Comparison of HBeAg positive patients and HBeAg negative ones indicated higher perforin level in the latter group (Table 2). Furthermore our results showed that serum perforin level in 2 HBeAg positive patients who were seroconverted to HBeAg negative was markedly higher than other patients (Table 2). In our study serum perforin level in patients with active chronic hepatitis B who showed virologic response to treatment (n = 13) was higher than without virologic response (Table 3).

Correlation analysis showed no significant correlation between perforin level and viral load, HBs Ag quantitative, stage and grade of the disease. 

**Table 1.** Characteristics of the Study Population (Data are Expressed as Mean ± Standard Deviation)

Characteristics	Active Patients, Mean ± SD	Carrier Patients, Mean ± SD	P value ^a^	Healthy Volunteers, Mean ± SD
**Age, y**	34.95 ± 9.16	42.17 ± 12.44	0.03	27.84 ± 6.58
**Stage**	1.90 ± 1.21	N.A ^b^	N.A	N.A
**Grade**	7.05 ± 1.76	N.A	N.A	N.A
**HBSAg^**b**^Quant**	17×10^3 ^± 2×10^3^	833.47 ± 1285.10	< 0.001	N.A
**Viral load pre treatment**	1.5×10^8 ^± 3.7×10^8^	982.50 ± 1586.97	< 0.001	N.A
**Viral load post treatment**	6.1×10^5 ^± 2.3×10^6^	N.A	N.A	N.A
**ALT ^**b**^pre test**	77.75 ± 57.16	31.83 ± 17.69	< 0.001	N.A
**ALT post test**	43.45 ± 26.52	N.A	N.A	N.A
**AST ^**b**^pre test**	44.55 ± 23.38	26.20 ± 9.60	0.002	N.A
**AST post test**	31.55 ± 11.44	N.A	N.A	N.A
**Perfor in level**	4.68 ± 2.29	2.63 ± 1.57	< 0.001	0.604 ± 0.379

^a^ P value base on independent sample t test or Mann-Whitney U test

^b^ Abbreviations ALT, Alanine Aminotransferase; AST, Aspartate Aminotransferase; HBSAg, Hepatitis B Surface Antigene; N.A, No Analyzed

**Table 2.** Frequency Distribution of Patients According to Sex, HBeAg Pretreatment,and HBeAg Post Treatment

Characteristics	No. (%)	Perforin Level
**Sex**		
male	15(75)	5.30
female	5(25)	2.80
**HBeAg pre treatment**		
positive	6(30)	N.A^a^
negative	14(70)	N.A
**HBeAg post treatment**		
positive	4(20)	3.46
negative	16(80)	4.98
**HBeAg seroconversion**		
negative	2(10)	7.23

^a^Abbreviation: N.A, No Analyzed

**Table 3.** Comparing Patients With and Without Virologic Response

Group of Patients	Viral Load	No.	Mean of Perforin Level	P value
**A** ^**a**^	Pre > 2000 and Post > 2000	5	3.40	0.135
**B ** ^**a**^	Pre > 2000 and Post < 2000	13	5.45	

^a^A, Under treatment patients without virologic response; B, Under treatment patients with virologic response

## 5. Discussion

About 1.5 million people in Iran are living with HBV infection (a mild to moderate prevalence according to the WHO classification), and it is assumed that from 15 to 40 percent of them are at risk of developing cirrhosis and/or hepatocellular carcinoma (HCC) without intervention (15). Chronic hepatitis B (CHB) infection can cause a spectrum of diseases ranging from clinically asymptomatic state to the development of cirrhosis-related complications and hepatocellular carcinoma (14). Perforin in the cellular immune response causes holes in infected cells, and the perforin pores can serve as passive conductor of granzymes and granulysin through the target cell membrane, and could also allow an ionic exchange, which causes an osmotic unbalance and in consequence, the cell death (16, 17). Perforin is an unstable molecule; therefore, the amounts vary according to the cytotoxic cell population (18). Large randomized controlled trials have confirmed the efficacy of PEG-INF in chronic hepatitis B (8). In accordance to the results, Kaser A et al. showed that stimulation with IFN-α increases perforin mRNA levels in PBMC (peripheral blood mononuclear cells), and based on their suggestion, upregulation of perforin by IFN-α results in elevated cytotoxicity, and have proposed that IFN-α might support elimination of virally infected cells via perforin pathway (19).

Understanding the immune response upon HBV infection is useful to develop appropriate therapeutic strategies for controlling viral hepatitis as well as improving current knowledge regarding hepatitis prognosis. Currently laboratory tests such as viral load, HBs Ag quantitative, liver enzyme measurement and etc. are used for monitoring response to PEG-IFN-alfa-2a treatment. Based on our results serum perforin level was higher in both groups of patients infected by HBV compared to the healthy volunteers. Furthermore serum perforin level was higher in patients with active chronic hepatitis B treated with PEG-IFN-α-2a than those with inactive chronic hepatitis B. Although the results were not statistically significant,which is probably due to low number of participants in this study. With reference to our results perforin is probably an important factor of immune system in dealing with hepatitis B infection. According to higher serum perforin level in patients under treatment with PEG-IFN-alfa-2a, there is this viewpoint that perforin measurement can be a tool in monitoring patients under treatment with PEG-IFN-alfa-2a, and can be used as a laboratory marker or a measure of response to treatment with PEG-IFN-alfa-2a. However further extensive research with larger number of patients is required to confirm the above findings and claims.

The most important limitation of the present study was those patients who withdrew from the treatment or left the study due to hypersensitivity to PEG-IFN-α. Another limitation of the study was time restriction.
